# Identification of the site of oxidase substrate binding in *Scytalidium thermophilum* catalase

**DOI:** 10.1107/S2059798318010628

**Published:** 2018-10-02

**Authors:** Yonca Yuzugullu Karakus, Gunce Goc, Sinem Balci, Briony A. Yorke, Chi H. Trinh, Michael J. McPherson, Arwen R. Pearson

**Affiliations:** a Department of Biology, Kocaeli University, Umuttepe, 41380 Kocaeli, Turkey; b The Hamburg Centre for Ultrafast Imaging, Institute for Nanostructure and Solid State Physics, Universität Hamburg, 22761 Hamburg, Germany; c Astbury Centre for Structural Molecular Biology, University of Leeds, Leeds LS2 9JT, England

**Keywords:** catalase, *Scytalidium thermophilum*, oxidase, 3-amino-1,2,4-triazole, NADPH, binding pocket, lateral channel

## Abstract

The catalase from *Scytalidium thermophilum* is a homotetramer containing a heme *d* in each active site. Although the enzyme has a classical monofunctional catalase fold, it also possesses oxidase activity towards a number of small organics, including catechol and phenol. In order to further investigate this, the crystal structure of the complex of the catalase with the classical catalase inhibitor 3-amino-1,2,4-triazole (3TR) was determined at 1.95 Å resolution. Surprisingly, no binding to the heme site was observed; instead, 3TR occupies a binding site corresponding to the NADPH-binding pocket in mammalian catalases at the entrance to a lateral channel leading to the heme. Kinetic analysis of site-directed mutants supports the assignment of this pocket as the binding site for oxidase substrates.

## Abstract 2

The structure of *Scytalidium thermophilum* catalase in complex with its well known inhibitor 3-amino-1,2,4-triazole revealed that the inhibitor occupies a surface pocket at the end of the lateral channel. This pocket corresponds to the site of NADPH binding in mammalian catalases. Peroxide-independent phenolic substrate oxidation is likely to occur in a similar manner to NADPH oxidation.

## 1. Introduction   

Catalases (hydrogen-peroxide:hydrogen-peroxide oxido­reductases; EC 1.11.1.6) are redox enzymes that are responsible for the dismutation of hydrogen peroxide into water and molecular oxygen (Loewen, 1999 ▸). They are found in almost all aerobic organisms and play a crucial role in prokaryotic and eukaryotic cell detoxification (Maté *et al.*, 2001 ▸). The crystal structures of 15 heme catalases, including that from the thermophilic fungus *Scytalidium thermophilum* (Yuzugullu *et al.*, 2013 ▸), have been solved at high resolution (Díaz *et al.*, 2012 ▸). The structures reveal a homotetrameric enzyme in which each of the four active sites consists of a pentacoordinated iron protoporphyrin IX prosthetic group with a tyrosin­ate axial ligand (Díaz *et al.*, 2012 ▸; Yuzugullu *et al.*, 2013 ▸). Some catalases also contain an NADPH cofactor tightly bound at the periphery of each subunit (Díaz *et al.*, 2012 ▸).

In the resting state the heme is in a high-spin ferric state (Fe^3+^), which is converted to compound I in a two-electron oxidation by hydrogen peroxide. One electron is removed from the Fe atom, forming an oxyferryl moiety (Fe^4+^=O) with one O atom from the hydrogen peroxide molecule, and the second electron is removed from the porphyrin, resulting in a π–cation radical (1). Compound I is reduced back to the native (ferric) state by a second molecule of hydrogen peroxide (2). Alternatively, under low hydrogen peroxide conditions, compound I can be reduced to compound II (3), which can react with another H_2_O_2_ to give the inactive compound III (4). For NADPH-binding catalases, it has been proposed that the enzyme is protected against compound III formation by the NADPH preventing or rescuing compound II formation (Sevinc *et al.*, 1999 ▸; Putnam *et al.*, 2000 ▸; Nicholls, 2012 ▸).
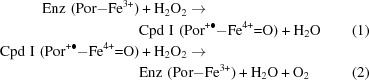


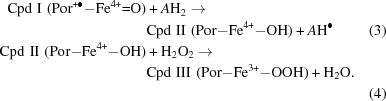



The heme is deeply buried inside the protein, with a complex network of channels providing access to the exterior (Sevinc *et al.*, 1999 ▸; Díaz *et al.*, 2012 ▸). The main channel approaches the distal side of the heme, perpendicular to the plane of the heme, and is the access route for hydrogen peroxide (Díaz *et al.*, 2012 ▸). A second channel, approaching the heme laterally, emerges on the enzyme surface at a location corresponding to the NADP(H)-binding pocket in catalases that bind a nicotinamide cofactor. For homologues that do not bind NADPH, there is some evidence that the channel is involved in either the exit of the reaction products (Díaz *et al.*, 2012 ▸) or the entry of substrates/inhibitors (Sevinc *et al.*, 1999 ▸). A third channel leading from the distal side of the heme to the central cavity of the tetramer is proposed to play a role in the oxidation of heme *b* to heme *d* for catalases that possess heme *d* in their active site (Murshudov *et al.*, 1996 ▸; Sevinc *et al.*, 1999 ▸; Putnam *et al.*, 2000 ▸), but no functional role has yet been presented for heme *b* catalases (Chelikani *et al.*, 2004 ▸).

We have previously shown that in addition to catalase activity, the catalase from *S. thermophilum* (CATPO) possesses a promiscuous phenolic oxidase activity in the absence of hydrogen peroxide (Ögel *et al.*, 2006 ▸; Sutay Kocabas *et al.*, 2008 ▸; Yuzugullu *et al.*, 2013 ▸). This peroxide-independent secondary activity of catalases has also been identified in other catalases (Vetrano *et al.*, 2005 ▸; Koclar Avci *et al.*, 2013 ▸; Lončar & Fraaije, 2015 ▸; Teng *et al.*, 2016 ▸) and has been presumed to also occur at the heme active site. Here, we report a combined structural, spectroscopic and kinetic analysis of CATPO that allows us to propose an alternative model.

## 2. Experimental procedures   

### 2.1. Materials   

Standard chemicals and biochemicals were obtained from Sigma and Merck. Molecular-size markers and DNA ladders were obtained from Bio-Rad and Biolab, respectively. Site-directed mutagenesis was performed using the QuikChange approach (Agilent).

### 2.2. Strains, plasmids and growth conditions   

*Escherichia coli* XL1-Blue (Stratagene) and BL21 Star (DE3) (Invitrogen) strains were used for cloning and expression, respectively. During cloning steps, *E. coli* cells were grown aerobically at 37°C in LB medium supplemented with 50 µg ml^−1^ kanamycin. The plasmid pET28a-CATPO (Yuzugullu *et al.*, 2013 ▸), which carries an N-terminal 6×His-tag sequence and TEV protease cleavage site, was used as the source of the *catpo* gene.

### 2.3. Site-directed mutagenesis   

Single-point mutations were introduced into the *catpo* coding region by QuikChange mutagenesis using Hot Start KOD DNA polymerase (Sigma). The PCR primers containing the desired mutations were purchased from Sentegen, Turkey and are listed in Table 1 ▸. Subsequent expression and purification were carried out as described previously (Yuzugullu *et al.*, 2013 ▸).

### 2.4. Enzyme assays   

Catalase and phenol oxidase activities were determined as described previously (Yuzugullu *et al.*, 2013 ▸). One unit of catalase was defined as the amount of enzyme that catalyses the decomposition of 1 µmol H_2_O_2_ per minute in a 10 m*M* H_2_O_2_ solution. The initial rates of H_2_O_2_ decomposition were used to determine the turnover number (*k*_cat_) and the apparent *K*_m_ values. Kinetic constants were derived by fitting *v versus* [S] traces to the Michaelis–Menten equation using *SigmaPlot* 14.0 (Systat Software Inc.). The term ‘*K*_m_app_’ in the context of catalases is the peroxide concentration at *V*_max_/2 and is used because the catalase reaction does not saturate with substrate and therefore does not precisely follow Michaelis–Menten kinetics (Switala & Loewen, 2002 ▸). One unit of phenol oxidase was defined as the amount of enzyme that catalyses the formation of one nanomole of product per minute. The effects of 3-amino-1,2,4-triazole (3TR) and catechol on oxidase activity were also investigated. Experiments with these compounds were conducted in the same manner but in the presence of the inhibitor at stated concentrations in the reaction buffer. Protein concentration was estimated using the Bradford assay (Bradford, 1976 ▸). All assays were performed in triplicate in 100 m*M* sodium phosphate buffer pH 7.0 at 60°C using a temperature-controlled spectrophoto­meter (Agilent Cary 50 or 60).

### 2.5. Crystallization, data collection and refinement   

Crystals were obtained by hanging-drop vapor diffusion using a reservoir consisting of 6–16%(*v*/*v*) PEG 400, 0.2 *M* potassium chloride, 0.01 *M* calcium chloride, 0.05 *M* sodium cacodylate in the pH interval 5.0–5.6. The complex of 3TR with CATPO was prepared by soaking crystals for 20 min in mother liquor containing 40 m*M* 3TR. Crystals were flash-cooled in liquid nitrogen (Teng, 1990 ▸) after soaking for several minutes in a synthetic mother liquor containing 20%(*v*/*v*) PEG 400 as a cryoprotectant. Diffraction data were collected on beamlines ID29 and ID30B at the European Synchrotron Radiation Facility (ESRF; de Sanctis *et al.*, 2012 ▸; McCarthy *et al.*, 2018 ▸) and on beamline I03 at Diamond Light Source (DLS; Allan *et al.*, 2015 ▸) at 100 K (Table 2 ▸) and were processed using *XDS* (Kabsch, 2010 ▸). Subsequent scaling (Evans, 1997 ▸), structure-solution, model-building and refinement steps were carried out using the *CCP*4 suite (Winn *et al.*, 2011 ▸). Although the E316F mutant data extended to higher resolution, refinement was unstable. Examination of the data using *AUSPEX* (Thorn *et al.*, 2017 ▸) showed a severe ice ring at ∼2.2 Å resolution. Truncation of the data set to 2.3 Å resolution resulted in stable refinement. The wild-type structure of CATPO (PDB entry 4aum; Yuzugullu *et al.*, 2013 ▸) was used to obtain initial phases by molecular replacement using *MOLREP* (Vagin & Teplyakov, 2010 ▸). Iterative model building and refinement were performed using *Coot* (Emsley *et al.*, 2010 ▸) and *REFMAC*5 (Murshudov *et al.*, 2011 ▸), with each chain treated as a single TLS domain (Winn *et al.*, 2001 ▸) and local NCS restraints (Usón *et al.*, 1999 ▸; Murshudov *et al.*, 2011 ▸).

The final structures of the E316F, H246W and V536W variants and the CATPO–3TR complex were determined at 2.3, 1.9, 1.8 and 1.91 Å resolution, respectively. The asymmetric units of the four CATPO variants analysed in this study each contained a CATPO homotetramer. The N-terminal 20 residues of all subunits in each of the four variants were disordered, as in the wild-type enzyme (Yuzugullu *et al.*, 2013 ▸), and were not included in the refined structures. The structure factors and coordinates for each structure have been deposited in the Protein Data Bank with accession codes 5y17 for the E316F mutant, 5xvz for the H246W mutant, 5xy4 for the V536W mutant and 5zz1 for the CATPO–3TR complex. All figures were prepared using *PyMOL* (http://www.pymol.org/).

## 3. Results and discussion   

**Figure 1:**
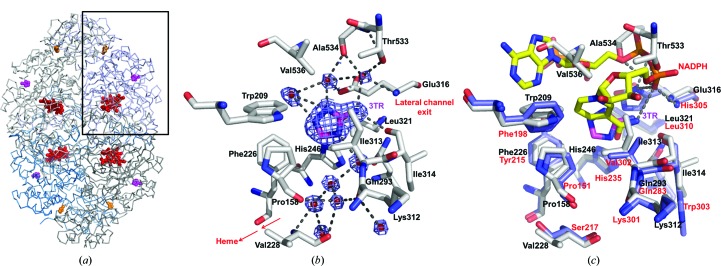
(*a*) The CATPO tetramer shown as a ribbon diagram, highlighting the heme and 3TR binding sites. The heme is colored red, 3TR in the oxidase pocket is colored pink and 3TR at the dimer interface is colored orange. (*b*) The 3TR binding site in the lateral channel of CATPO. Composite OMIT electron density, calculated using the *CCP*4 *COMIT* program (Winn *et al.*, 2011 ▸), for 3TR and bound waters is drawn at 1 r.m.s.d. and shown as a blue wire mesh. Analysis of the hydrogen bonding suggests that 3TR is bound as 2*H*-1,2,4-triazole-3-amine and at the pH of the crystals should be in its neutral form. (*c*) View of chain *A* of the CATPO complex with 3TR (PDB entry 5zz1; grey) superposed onto human catalase (PDB entry 1dgh; blue). CATPO loop 533–537 lies across the top of the NADPH-binding pocket, clashing with the position of the NADPH in the human enzyme.

Despite extensive efforts, we have been unable to obtain a crystal of CATPO in complex with catechol owing to its rapid auto-oxidation at concentrations high enough for binding, given the low *K*_m_. However, we were able to obtain the structure of its complex with the widely used catalase inhibitor 3-amino-1,2,4-triazole (3TR; Margoliash *et al.*, 1960 ▸; Nicholls, 1962 ▸) to 1.91 Å resolution (Table 2 ▸). Surprisingly, and in contrast to other structural reports of complexes of 3TR with catalase [Putnam *et al.*, 2000 ▸; Borovik *et al.*, 2011 ▸; PDB entry 1th4 (R. Sugadev, M. N. Ponnuswamy, D. Kumaran, S. Swaminathan & K. Sekar, unpublished work)], we did not observe 3TR bound at the heme (Fig. 1 ▸ *a*). Instead, 3TR occupies a surface pocket at the end of the lateral channel leading from the heme, where its interactions with the protein are almost exclusively mediated by a series of well ordered water molecules (Fig. 1 ▸ *b*), as well as a second binding site at the interdimer interfaces of the homotetramer (Fig. 1 ▸ *a*). Interestingly, the pocket at the end of the lateral channel corresponds to the site of NADPH binding in mammalian catalases (Fig. 1 ▸ *c*). However, CATPO contains a C-terminal extension, residues 533–537, that does not exist in the NADPH-binding catalases and that lies across the upper region of this pocket, preventing the binding of NADPH. The mediation of the 3TR–CATPO interaction by water molecules in the plane of the inhibitor suggests a flexible binding site with the possibility of accommodating a variety of planar ligands of different sizes, suggesting that this could also be the site of phenolic substrate binding.

**Figure 2:**
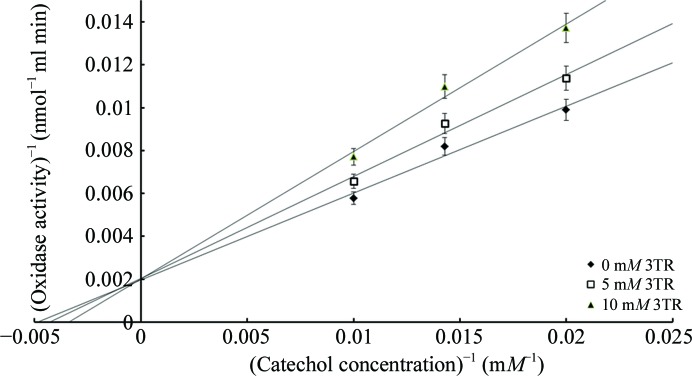
An illustrative double-reciprocal plot (Lineweaver & Burk, 1934 ▸) is presented showing classical competitive inhibition kinetics for 3TR with respect to the CATPO oxidase activity. Error bars show the standard deviation of the *SigmaPlot* fit of the raw data for each point. Full details of the analysis are provided in Supplementary Fig. S1.

To further investigate this possibility, oxidase assays were performed at increasing concentrations of the CATPO oxidase substrate catechol (0–300 m*M*) with and without 3TR (0–10 m*M*). The *K*_m_app_ and *V*_max_ values for catechol were calculated as 92.5 m*M* and 12 500 nmol ml^−1^ min^−1^, respectively. 3TR showed competitive inhibition with respect to catechol, with a *K*_i_ of 2.1 × 10^−2^ *M* (Fig. 2 ▸). This indicates that 3TR and catechol bind at the same site, but does not allow us to conclude that this site is the 3TR binding site observed crystallographically. We therefore generated a series of site-directed mutants around the putative 3TR/catechol binding pocket to further test this hypothesis.

The residues Pro158, His246, Gln293, Ile313, Ile314, Glu316, Leu321 and Val536 surround the 3TR binding pocket and are structurally homologous to the residues that bind NADPH in the mammalian catalases. They were individually mutated to residues of the opposite size (*i.e.* small to large and *vice versa*) and the resulting variants were characterized. Specifically, the variants P158W, H246W, Q293W, I313F, I314F, E316F, E316H, L321A, V536A and V536W were constructed. All except P158W and Q293W were expressed normally. The catalase turnover numbers (*k*_cat_) are essentially unaffected for the E316F, E316H, H246W, I313F, I314F and L321A variants, but a marked increase in the catalase *k*_cat_ value was observed for V536W, accompanied by a large increase in *K*_m_app_ for H_2_O_2_ (Table 3 ▸). The catechol oxidase activities of the H246W, I314F, L321A and V536W variants were noticeably reduced (50–92%) with respect to the wild-type enzyme, whereas the E316F, E316H, I313F and V536A variants of CATPO had little effect on the oxidase activity.

**Figure 3:**
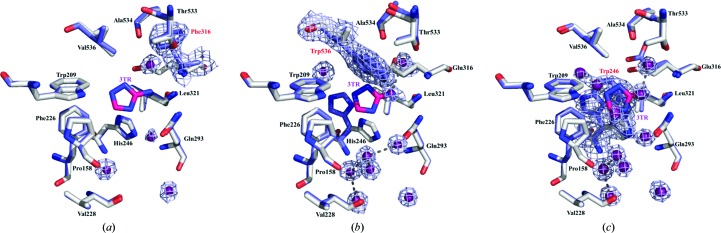
Comparison of 3TR binding sites in the lateral channel of the E316F (*a*), V536W (*b*) and H246W (*c*) variants superposed onto the complex of CATPO with 3TR. The corresponding 2*F*_o_ − *F*_c_ electron density, contoured at 0.7 r.m.s.d., is shown for the three cases as a blue mesh. Changes in solvent organization are evident among the structures. Trp246 has two alternate conformations (Fig. 4 ▸).

To further probe the effect of the mutations, the crystal structures of three variants were determined: H246W (lowest oxidase activity), E316F (little effect on oxidase activity) and V536W (40% reduced oxidase activity, but a major effect on catalase kinetic parameters) (Table 3 ▸). The resulting structures were almost identical to the native enzyme (Table 2 ▸), aside from changes in the ordered solvent molecules found in the putative oxidase substrate-binding pocket, the lateral channel and the main channel through which peroxide is thought to reach the heme active site. Surprisingly, the most extreme example of this is in the E316F mutant, in which the upper main channel and the outer part of the lateral channel contain almost no ordered solvent molecules; however, this is likely to be at least partially attributable to the lower resolution of this data set (Figs. 3 ▸ *a* and 4 ▸ *b*). The bulky phenylalanine side chain of the E316F variant is oriented away from the entrance to the lateral channel and putative oxidase substrate-binding pocket and thus it is not surprising that mutations at this position show minimal effect on the oxidase activity. In contrast, the bulky tryptophan residue of the V536W variant protrudes into the top of the putative oxidase substrate site, partially occluding it, consistent with the partial reduction in oxidase activity (Figs. 3 ▸ *b* and 4 ▸ *d*).

A similar picture is seen for His246, which lies directly below and perpendicular to the 3TR ring in the CATPO–3TR complex. In the H246W variant both of the alternate conformations of the tryptophan side chain that were observed protrude further into the 3TR binding site and are likely to considerably hinder the binding of any small organic substrate (Figs. 3 ▸ *c* and 4 ▸ *c*). This is consistent with the marked reduction in oxidase activity for this mutant. Interestingly, in the V536W variant, although not in the wild-type enzyme, His246 also adopts an alternate conformation, although this does not protrude into the 3TR binding site (Figs. 3 ▸ *b* and 4 ▸ *d*). Both the V536W and H246W variants therefore support our assignment of this pocket as the oxidase substrate-binding site.

**Figure 4:**
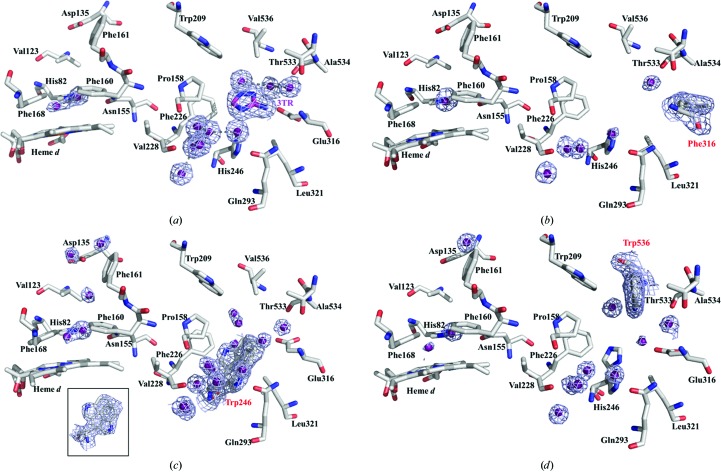
Main and lateral channel solvent in CATPO–3TR (*a*) and the E316F (*b*), H246W (*c*) and V536W (*d*) variants. The corresponding electron densities are shown for the four cases. Possible hydrogen-bond interactions are shown as dashed lines. Lys312, Ile313 and Ile314 in the lateral channel were removed for clarity. Mutated residues are shown in red and the ligand 3TR in purple. The inset in (*c*) shows the two alternate conformations of Trp246 face on with density. 2*F*_o_ − *F*_c_ electron density is shown as a blue mesh contoured at 0.7 r.m.s.d.. The channels are shown as transparent surfaces in Supplementary Fig. S2.

Val536 is part of the C-terminal extension of CATPO that blocks the upper part of the NADPH-binding pocket in mammalian catalases and thus it was surprising that changes in this residue had such marked effects on the catalase activity. As for the E316F variant, in the V536W variant the chain of ordered solvent molecules in the main channel is disrupted (Fig. 4 ▸ *d*), suggesting that there may be some crosstalk between the main and lateral channels. However, in contrast to the V536W mutation, the E316F mutation has almost no effect on the catalase activity (Table 3 ▸). In addition, there is no obvious structural change or alteration in relative *B*-factor distribution in either mutant compared with the apo wild-type structure (PDB entry 4aum; Yuzugullu *et al.*, 2013 ▸) that could explain this observation, and further investigation will be required.

**Figure 5:**
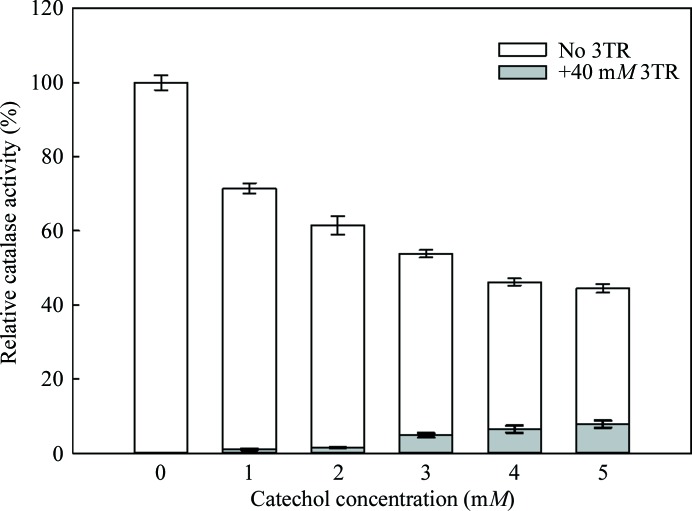
The effect of increasing catechol concentrations (up to 5 m*M*) on the inhibition of CATPO by 3TR at a constant concentration of 40 m*M*.

The currently accepted model for 3TR inhibition of catalases is *via* either a reversible binding mode at high 3TR concentration (Appleman *et al.*, 1956 ▸; Margoliash *et al.*, 1960 ▸) or a slow peroxide-dependent irreversible inactivation that results in the formation of a covalent adduct at the heme active site (Borovik *et al.*, 2011 ▸). The covalent adduct is likely to be the state that was observed in the previously reported complexes of 3TR with other catalases (Putnam *et al.*, 2000 ▸; Borovik *et al.*, 2011 ▸; PDB entry 1th4). However, the 3TR binding site for reversible inhibition has not been identified to date, although its noncompetitive nature would be consistent with a non-heme-centred mode of action (Nicholls, 1962 ▸; Putnam *et al.*, 2000 ▸). We therefore wondered whether this reversible inhibition could in fact be mediated *via* the oxidase substrate-binding site that we have identified. In this case, we would predict that catechol binding would protect the CATPO catalase activity against 3TR inhibition. We therefore carried out a competition assay at a constant concentration of 3TR (40 m*M*) that is sufficient to nearly totally inhibit the catalase activity and increasing amounts of catechol, and observed that the presence of catechol reduces 3TR inhibition in a dose-dependent manner (Fig. 5 ▸). Interestingly, catechol itself inhibits the catalase activity, but in a much less potent fashion than 3TR. This suggests that, as proposed by others (Nicholls, 1962 ▸; Putnam *et al.*, 2000 ▸), the reversible inhibition by 3TR is mediated *via* an allosteric effect and that other molecules binding to this pocket will also have an inhibitory action. In this context it is interesting to note that the 3TR complex also shows a reduction in the number of ordered water molecules in the main channel when compared with the apo wild-type structure (Supplementary Fig. S3).

## 4. Conclusions   

In summary, based on our structural, mutation and kinetic data we propose that the pocket at the entrance to the lateral channel, occupied by the nicotinamide moiety of NADPH in mammalian catalases, is the site of both oxidase substrate and 3TR binding. The promiscuous nature of the CATPO oxidase is explained by the presence of a number of ordered water molecules that both mediate substrate binding by forming bridging hydrogen bonds and can be displaced to accommodate different sized and shaped substrates. Peroxide-independent phenolic substrate oxidation is then likely to occur in a similar manner to NADPH oxidation, *via* electron transfer from the substrate to a high-valent iron–oxo intermediate, presumably formed *via* reaction with oxygen.

## Supplementary Material

PDB reference: CATPO, H246W mutant, 5xvz

PDB reference: V536W mutant, 5xy4

PDB reference: E316F mutant, 5y17

PDB reference: CATPO–3TR complex, 5zz1

PDB reference: CATPO, 4aum

Suplementary Figures.. DOI: 10.1107/S2059798318010628/ud5005sup1.pdf

## Figures and Tables

**Table 1:** Oligonucleotides used in site-directed mutagenesis of *catpo*

Mutant	Sequence change	Oligonucleotide†
E316F	GAA→TTC	5′-CCGACCAAAATCATCCCGTTCGAATACGCTCCGCTGACC-3′
E316H	GAA→CAC	5′-CCGACCAAAATCATCCCGCACGAATACGCTCCGCTGACC-3′
H246W	CAC→TGG	5′-CTGATCAAATGGTGGTTCAAATCTCGTCAGGGTAAAGCTAGTCTGG-3′
I313F	ATC→TTC	5′-GGACCCGACCAAATTCATCCCGGAAGAATACGC-3′
I314F	ATC→TTC	5′-GGACCCGACCAAAATCTTCCCGGAAGAATACGC-3′
L321A	CTG→GCG	5′-CCGGAAGAATACGCTCCGGCGACCAAACTGGGTCTG-3′
P158W	CCG→TGG	5′-CGTTGGTAACAACATCTGGGTTTTCTTCATCCAGGACGC-3′
Q293W	CAG→TGG	5′-GGGACGTATGCGTATGGATCGTTGACGAATCTCAGGC-3′
V536A	GTT→GCG	5′-CAAAACCGCTGGTGCGTCTATCGTTGGTTCTGG-3′
V536W	GTT→TGG	5′-CAAAACCGCTGGTTGGTCTATCGTTGGTTCTGGTCCG-3′

† The underlined sequence is the codon that has been modified.

**Table 2:** Crystallographic data-collection and refinement statistics Values in parentheses are for the outermost shell.

	E316F variant	H246W variant	V536W variant	3TR complex
PDB code	5y17	5xvz	5xy4	5zz1
Beamline	ID30B, ESRF	ID30B, ESRF	ID30B, ESRF	I03, DLS
Detector	PILATUS3 6M	PILATUS3 6M	PILATUS3 6M	ADSC Q315
Oscillation angle (°)	0.1	0.05	0.05	0.5
Exposure time (s)	0.02	0.02	0.02	0.4
Transmission (%)	22	11	13	50
No. of images	1140	2500	1860	720
Wavelength (Å)	0.98	0.98	0.98	1.0
Space group	*I*2	*I*2	*I*2	*I*2
Unit-cell parameters				
*a* (Å)	125.7	125.3	125.4	125.5
*b* (Å)	120.9	120.8	120.7	121.7
*c* (Å)	183.8	185.2	184.7	185.5
β (°)	102.0	102.0	102.0	102.2
Resolution (Å)	100.3–2.3 (2.34–2.30)	100.5–1.9 (1.93–1.90)	90.3–1.8 (1.83–1.80)	29.4–1.91 (1.95–1.91)
Mosaicity (°)	0.24	0.08	0.11	0.17
*R*_merge_ † (%)	6.8 (27.4)	6.9 (45.6)	5.4 (52.0)	5.7 (42.6)
*R*_p.i.m._ ‡ (%)	5.2 (20.9)	4.7 (35.7)	4.7 (45.5)	3.7 (23.5)
CC_1/2_	0.996 (0.941)	0.998 (0.740)	0.998 (0.643)	0.998 (0.843)
Observed reflections	236441 (12331)	493165 (21545)	419138 (20025)	690894 (30098)
Unique reflections	105712 (5468)	200277 (9502)	223187 (11150)	206818 (9578)
Completeness (%)	88.8 (92.7)	94.5 (90.9)	89.9 (91.1)	98.8 (92.5)
Multiplicity	2.2 (2.3)	2.5 (2.3)	1.9 (1.8)	3.3 (3.1)
〈*I*/σ(*I*)〉	8.4 (3.1)	6.6 (1.8)	8.8 (1.6)	10.2 (2.7)
Refinement				
*R*_work_ (%)	17.8 (24.5)	16.2 (31.2)	15.8 (30.0)	14.0 (20.4)
*R*_free_ § (%)	22.0 (28.6)	19.1 (31.2)	19.3 (33.6)	16.5 (22.0)
No. of protein atoms	21079	21452	21421	21337
No. of solvent molecules	925	1837	2031	1618
No. of ligand atoms	176	236	183	224
No. of ion atoms	10	12	10	5
Average *B* factor (Å^2^)				
Protein	33.99	24.45	19.85	20.91
Ligands	24.44	27.68	19.08	16.02
Solvent	27.39	28.29	31.93	23.63
Ions	43.07	43.08	31.29	26.63
R.m.s.d., bond lengths¶ (Å)	0.0121	0.0147	0.0140	0.0150
R.m.s.d., bond angles¶ (°)	1.651	1.818	1.727	1.888
Ramachandran plot††				
Most favoured regions (%)	96.65	98.03	96.82	97.48
Outliers (%)	0.71	0	0.63	0.55
Alignment with wild-type structure‡‡ (PDB entry 4aum; Yuzugullu *et al.*, 2013 ▸) over all residues				
R.m.s.d. (Å)	0.232	0.226	0.242	0.242
*Q*-score	0.984	0.985	0.980	0.980

† *R*_merge_ = 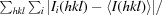 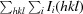.

‡ *R*_p.i.m._ is the precision-indicating (multiplicity-weighted) *R*_merge_ relative to *I* ^+^ or *I* ^−^.

§ *R*_free_ was calculated with 5% of the reflections that were set aside randomly.

¶ Based on the ideal geometry values of Engh & Huber (1991 ▸).

†† Ramachandran analysis using *MolProbity* (Chen *et al.*, 2010 ▸).

‡‡ R.m.s.d and *Q*-scores were calculated using *GESAMT* (Krissinel, 2012 ▸)

**Table 3:** Kinetic constants

Variant	*K*_m_app_ † (m*M*)	*k*_cat_ (s^−1^)	*k*_cat_/*K*_m_app_ (s^−1^ *M* ^−1^)	*R*_Z_ ‡	Heme type	Specific oxidase activity (nmol mg^−1^ min^−1^)
CATPO	10	203410	20.3 × 10^6^	0.8	*d*	213 ± 5
E316F	20	196000	9.8 × 10^6^	0.8	*d*	173 ± 2
E316H	33	261000	7.9 × 10^6^	0.8	*d*	163 ± 2
H246W	40	152000	3.8 × 10^6^	0.8	*d*	33 ± 2
I313F	10	205000	20.5 × 10^6^	0.9	*d*	232 ± 16
I314F	50	194000	3.9 × 10^6^	0.8	*d*	92 ± 7
L321A	11	274000	24.3 × 10^6^	0.9	*d*	107 ± 1
V536A	67	860500	12.9 × 10^6^	0.8	*d*	236 ± 6
V536W	600	2402000	4.0 × 10^6^	0.6	*d*	85 ± 7

† *K*_m_app_ is the H_2_O_2_ concentration at *V*_max_/2 and is used because the catalase reaction does not saturate with substrate and therefore does not precisely follow Michaelis–Menten kinetics (Switala & Loewen, 2002 ▸).

‡ *R*_Z_ = *A*_406_/*A*_280_.
