# Supplementary material for: Identification of the site of oxidase substrate binding in Scytalidium thermophilum catalase
Source: Acta Crystallogr D Struct Biol. 2018 Oct 2;74(Pt 10):979–85. doi: 10.1107/S2059798318010628 (PMC6173053; doi:10.1107/S2059798318010628)
Supplement: Supplementary file 1 [file d-74-00979-sup1.pdf]

STRUCTURAL
BIOLOGY

Volume 74 (2018)

Supporting information for article:

Identification of the site of oxidase substrate binding in the *Scytalidium thermophilum* catalase

Yonca Yuzugullu Karakus, Gunce Goc, Sinem Balci, Briony A. Yorke, Chi H. Trinh, Michael J. McPherson and Arwen R. Pearson

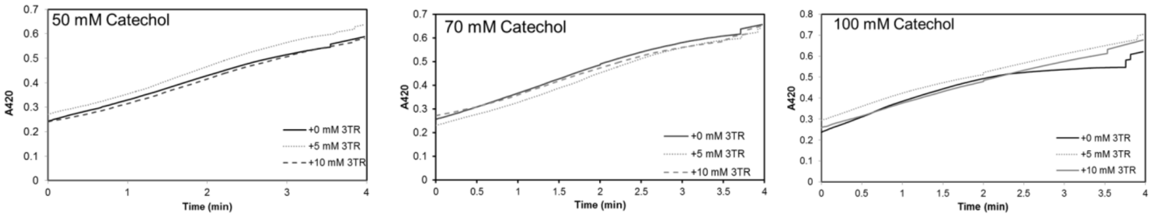

(a)

	3TR concentration		
	0 mM	5 mM	10 mM
Catechol concentration (mM)	Initial oxidase activity (nmole ml^{-1} min^{-1})		
50	101±5	88±4	73±3
70	130±6	114±4	96±4
100	172±7	152±7	130±6

(b)

3TR concentration (mM)	K_{M_app} (mM)	V_{max} (nmole ml^{-1} min^{-1})
0	243±2	588±23
5	286±3	588±28
10	357±3	588±28

(c)

Figure S1 *(a)* The oxidase activity of catalase monitored by changes in absorbance for 50, 70 and 100 mM Catechol *(b)* Table showing initial rates observed in the presence of catechol (50, 70 and 100 mM) and aminotriazole (3TR; 0, 5 and 10 mM) *(c)* Table showing K_M and V_{max} values measured in the presence of aminotriazole (3TR; 0, 5 and 10 mM)

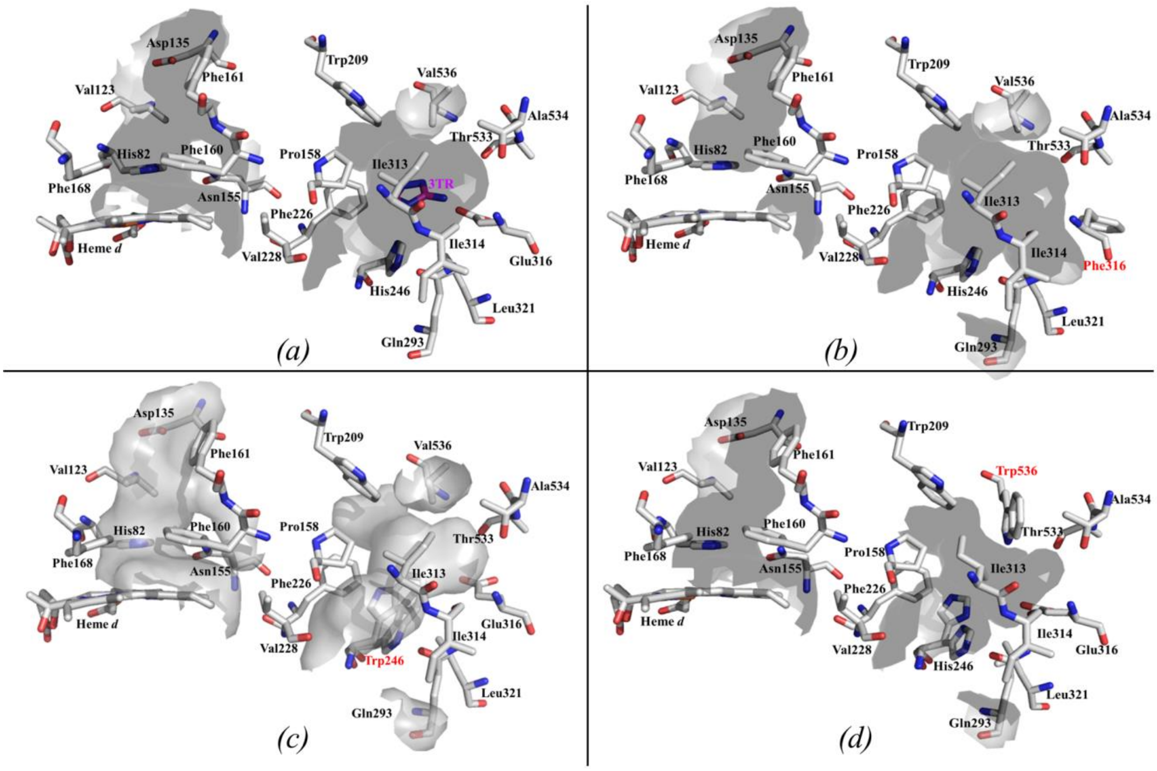

Figure S2 Comparison of main and lateral channels of for CATPO-3TR *(a)*, E316F *(b)*, H246W *(c)* and V536W *(d)*. The channels are presented with a transparent surface for the four cases. Mutated residues are given in red, the ligand 3TR purple.

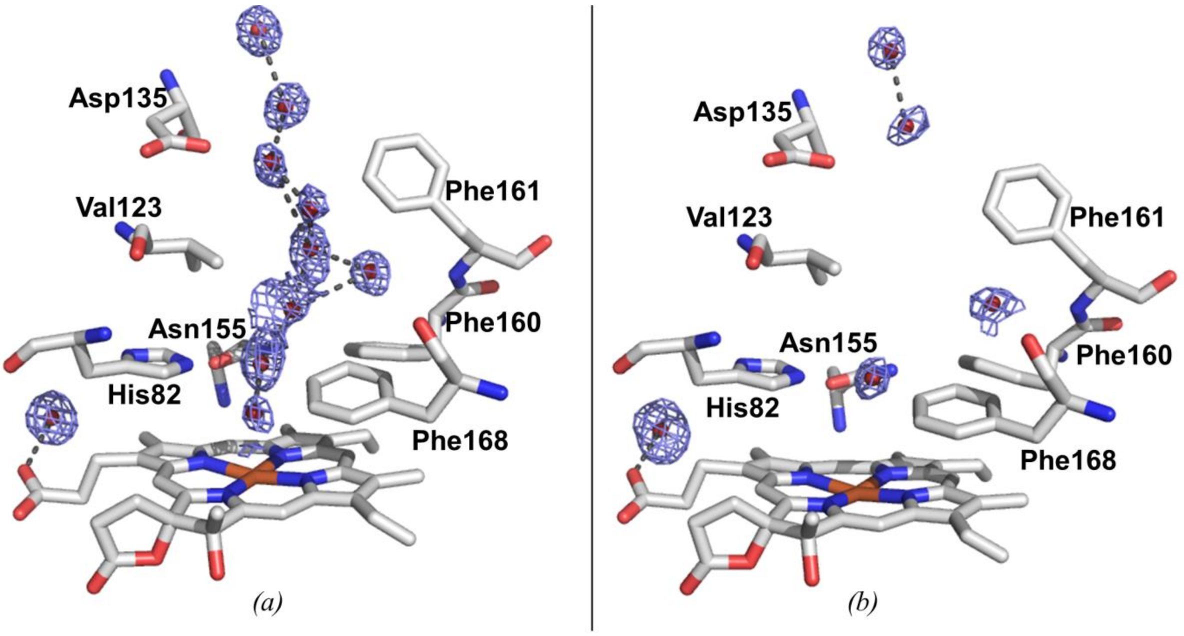

Figure S3 Comparison of main channel solvent for wild type CATPO (PDB 4AUM, Yuzugullu et al., 2013) *(a)* and CATPO-3TR *(b)*. The corresponding 2Fo-Fc electron density is drawn at 0.7 r.m.s.d as blue mesh. For clarity, only the catalytically important residues His82, Asn155, and Phe168 on the heme distal side are explicitly shown. Also displayed are the conserved residues lining the channel, Val123, Asp135, Phe160, and Phe168.
